# Neural Correlates of Opposing Effects of Emotional Distraction on Perception and Episodic Memory: An Event-Related fMRI Investigation

**DOI:** 10.3389/fnint.2012.00070

**Published:** 2012-09-19

**Authors:** Andrea T. Shafer, Florin Dolcos

**Affiliations:** ^1^Centre for Neuroscience, University of AlbertaEdmonton, AB, Canada; ^2^Department of Psychology, Beckman Institute for Advanced Science and Technology, Neuroscience Program, University of IllinoisUrbana-Champaign, IL, USA

**Keywords:** affect, emotional automaticity, emotional distraction, emotional memory, encoding success

## Abstract

A main question in emotion and memory literature concerns the relationship between the immediate impact of emotional distraction on perception and the long-term impact of emotion on memory. While previous research shows both automatic and resource-mediated mechanisms to be involved in initial emotion processing and memory, it remains unclear what the exact relationship between the immediate and long-term effects is, and how this relationship may change as a function of manipulations at perception favoring the engagement of either more automatic or mediated mechanisms. Using event-related functional magnetic resonance imaging, we varied the degree of resource availability for processing task-irrelevant emotional information, to determine how the initial (impairing) impact of emotional distraction related to the long-term (enhancing) impact of emotion on memory. Results showed that a direct relationship between emotional distraction and memory was dependent on automatic mechanisms, as this was found only under conditions of limited resource availability and engagement of amygdala (AMY)-hippocampal (HC) mechanisms to both impairing and enhancing effects. A hemispheric disassociation was also identified in AMY-HC, where while both sides were associated with emotional distraction and left AMY and anterior HC were linked to emotional memory, functional asymmetry was only identified in the posterior HC, with only the left side contributing to emotional memory. Finally, areas dissociating between the two opposing effects included the medial frontal, precentral, superior temporal, and middle occipital gyri (linked to emotional distraction), and the superior parietal cortex (linked to emotional memory). These findings demonstrate the relationship between emotional distraction and memory is context dependent and that specific brain regions may be more or less susceptible to the direction of emotional modulation (increased or decreased), depending on the task manipulation, and processes investigated.

## Introduction

An important question in the emotion literature concerns the relationship between the immediate impact of emotional distraction on perception and the long-term impact of emotion on memory. Typically, in the context of distraction and dual task paradigms, task-concurrent emotional distraction impairs task-relevant performance as the emotional information tends to capture and reallocate cognitive resources (Vuilleumier et al., 2001; Kensinger and Corkin, 2003; Mitchell et al., 2007; Talmi et al., 2007; Hodsoll et al., 2011; Pottage and Schaefer, 2012). This has been thought to occur as a result of privileged processing for emotional information, due to its increased relevance for survival. It is not clear, however, how this initial processing of distracting emotional information influences memory for the distracters themselves, and what the neural mechanisms linking the immediate and long-term effects of distracting emotions are. The present study addressed this issue using functional magnetic resonance imaging (fMRI) and an experimental design that assessed both the immediate (*impairing*) and long-term (*enhancing*) effects of task-irrelevant emotional distraction.

The severity by which emotional distraction impacts perception has been shown to be influenced by two factors: the degree of cognitive demand or attentional resources required to perform the main task, and the degree of emotional challenge (Vuilleumier et al., 2001; Pessoa et al., 2002, 2005; Anderson et al., 2003; Vuilleumier, 2005; Mitchell et al., 2007; Silvert et al., 2007; Shafer et al., 2012). Previous research investigating these factors yielded mixed findings, consistent either with the view that emotion processing is automatic and independent of attentional resources (*traditional* view), or consistent with the view that emotion processing depends on manipulations that affect the availability of processing resources (*competing view*), linked to the demands/difficulty of the main task. However, these studies have not involved systematic manipulations of both task difficulty and emotional challenge. A recent study investigating this issue in a “lower-level” perceptual task that manipulated both of these factors provided evidence that processing of emotional distraction is both automatic and modulated by attention (Shafer et al., 2012), which is consistent with both views. Specifically, consistent with the traditional view, we found that overall emotional distraction impacted task performance regardless of the attentional demands necessary to perform the main task. However, consistent with the competing view, we also found that the highest level of disruption by emotional distraction occurred when most resources were available for distraction. These results suggest that two mechanisms contribute to the immediate impact of emotional distraction on perception: one rooted in automaticity and the other modulated by attention. What remains unclear is how these manipulations at perception affecting the immediate impact of emotion may also influence the long-term effects of emotion on memory.

Regarding the long-term impact of emotion on memory, extant evidence also suggests the existence of two routes contributing to the memory-enhancing effect of emotion (Dolcos et al., 2004a,b, 2011; Kensinger and Corkin, 2004; LaBar and Cabeza, 2006). One route, consisting of medial temporal lobe (MTL) structures comprised of emotion-based (amygdala, AMY) and memory-based (hippocampal structures, HC) regions, is thought to operate more automatically and largely independently of resources at the time of encoding. The other route, involving prefrontal and parietal cortices, is thought to depend on the contribution of other processes to the memory-enhancing effect of emotion, such as working memory, semantic memory, and attention. Evidence supporting the dissociation between the automatic and mediated routes has shown, for instance, that the AMY-HC engagement is associated with emotional memory following a shallow level of processing during encoding, whereas areas previously shown to be modulated by attention were more sensitive to emotional memory under a deep level of processing (Dolcos et al., 2004a,b, 2011; Ritchey et al., 2011). Overall, these results lend support to the idea that the memory-enhancing effect of emotion can result from both automatic and mediated/attention-dependent mechanisms.

A main open question concerns the relationship between the immediate and long-term effects of emotion in conditions where emotional information is presented as task-irrelevant distraction, especially given that both effects seem to engage automatic and mediated/attention-dependent mechanisms. Specifically, it is not clear whether there is a one-to-one relationship between the two opposing effects of task-irrelevant information on perception and memory – i.e., is there a direct link between the immediate (*impairing*) and long-term (*enhancing*) impact, such that the conditions in which emotional distraction produces the strongest immediate impact will also be translated in the strongest long-term impact on memory? If so, this would suggest that reallocation of processing resources by emotional distraction, overlapping with the initiation of processing leading to better memory for the distracters themselves, is the main mechanism linking the immediate/impairing and long-term/enhancing effects of task-irrelevant emotional information. Alternatively, it is possible that the link between the impairing and enhancing effects does not occur when the former effect is maximized, and hence would likely involve slightly different mechanisms.

Previous research investigating how immediate resource allocation relates to long-term memory via manipulations of the amount of resources allocated toward the to-be-remembered items has shown that divided attention at the time of encoding negatively influences how well those items will be remembered compared to items encoded with full/non-divided attention (Hicks and Marsh, 2000; Craik, 2001; Uncapher and Rugg, 2005, 2008). However, similar manipulations with emotional stimuli have shown smaller decrements in memory performance when attention was divided, although this resilience in memory came at a cost, as performance on the primary task was disrupted by the presence of emotional distraction (Kensinger and Corkin, 2003; Talmi et al., 2007; Pottage and Schaefer, 2012). Overall, these findings suggest a direct relationship between the immediate and long-term impact of emotional distraction, possibly involving automatic mechanisms, although a role of mediated attention-related mechanisms is also implied. It is not clear, however, what the circumstances are in which a direct link between the immediate (impairing) and long-term (enhancing) impact of emotion can be found, what the neural correlates of the link between these opposing effects are, and how they are distinguished from those involved in one (immediate/impairing) or the other (long-term/enhancing) of these effects.

The overarching goal of the current study was to investigate the relationship between the immediate (impairing) and long-term (enhancing) effects of emotion by (i) examining how emotionally distracting information at perception influences the memory-enhancing effect of emotion, and by (ii) identifying common and dissociable neural correlates of emotional distraction on perception and encoding success, thus linking the behavioral effects of emotional distraction and memory. These issues were investigated using a perception task involving manipulation of cognitive demand of goal-relevant processing in the presence of emotional distraction, followed by a surprise memory task for the distracters themselves, while event-related fMRI data were recorded.

Based on the extant evidence suggesting possible relationships between the immediate and long-term impact of emotional distraction, we made the following conditional predictions. First, regarding the behavioral effects, if there is a one-to-one relationship between the immediate/impairing and long-term/enhancing impact of emotion, we predict that the condition with the strongest immediate impact of emotion will produce the strongest long-term impact. Alternatively, if other factors also contribute to one or the other of these opposing effects, conditions where the immediate impact of emotion is present may not necessarily lead to a long-term impact of the same extent, and vice-versa. Regarding the neural correlates of these effects, if the same automatic and attention-mediated processes are involved in both the immediate and long-term effects and there is a one-to-one relationship between the two effects in the behavioral data, then we predict an overlap in the responses to the immediate and long-term impact of emotion in the same areas of the emotion network (e.g., AMY). However, if dissociable processes are involved in the immediate and long-term effects and there is no one-to-one relationship between the two effects, then we predict largely dissociable regions associated with the immediate and long-term effects of emotional distraction.

## Materials and Methods

### Participants

The present investigation involved analyses on data from 16 (seven males) healthy right-handed young adults (19–34 years), recruited from the University of Alberta and Edmonton City area. Participants signed an informed consent form before participating, and were reimbursed for their participation. The experimental protocol was approved for ethical treatment of human participants by the Health Research Ethics Board at the University of Alberta.

### Tasks and stimuli

**Figure 1:**
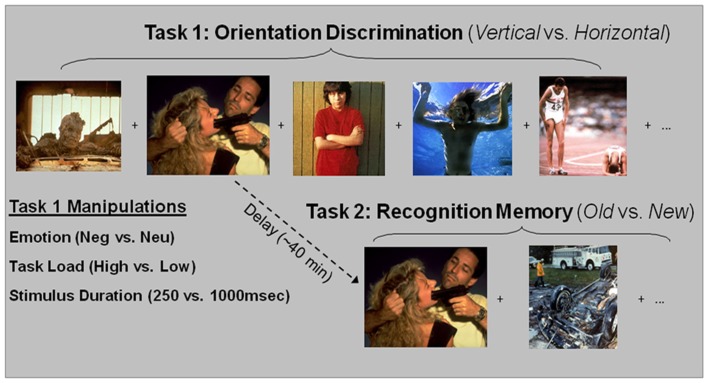
**Diagrams of perception and memory tasks**. Trial type during the orientation discrimination task was defined by the type of distraction in the rectangular picture (Emo, Neu), the duration of the stimulus (250, 1000 ms), and the perceptual load necessary to perform the task (High, Low). Participants were instructed to determine the orientation of the shape. Following the perception task, participants were given a surprise recognition memory task for a sub-set of the distracters presented in the perception task. Participants were instructed to determine if the pictures were from the perception task “Old” or were “New,” not presented during the perception task. Emo, Emotional; Neu, Neutral.

Participants completed two tasks, both performed in the scanner: a perceptual orientation discrimination task with distraction and an episodic memory task (see task diagram illustrated in Figure 1). In the perception task, participants made decisions on the orientation of vertical and horizontal pictures with negative and neutral content, and in the memory task they made decisions about whether emotional and neutral pictures were presented during the perception task or not. Since the focus of the current paper is on encoding success only fMRI data from the perception task were analyzed.

#### Perception task

The stimuli and design of the perception task were described in a previous report focusing on the perceptual task (Shafer et al., 2012). Briefly, the task used pictures selected from the International Affective Pictures System (Lang et al., 2008), based on their normative scores for arousal and valence and was supplemented with in-house pictures used in previous studies (Yamasaki et al., 2002; Dolcos and McCarthy, 2006). Distraction type was manipulated by the emotional content (negative vs. neutral) of the rectangular pictures. Attentional demand was manipulated by varying the presentation time of the stimuli (Short Dur = 250 ms vs. Long Dur = 1000 ms) and by varying the ratio of the horizontal vs. vertical sides of the rectangles (Lo-Load = clearly rectangles vs. Hi-Load = closer to squares). These two manipulations were chosen because both are considered manipulations of task demand (Grill-Spector and Kanwisher, 2005; LaBar and Cabeza, 2006), and to be consistent with research from both perception and memory domains. Specifically, a shorter presentation time (i.e., 250 ms) is consistent with investigations of the effect of processing load in studies of perception (e.g, Pessoa et al., 2002, 2005), while a longer presentation time (i.e., 1000 ms) is more consistent with paradigms investigating emotional memory (e.g., Ritchey et al., 2008). Participants were instructed to maintain focus on the orientation task and determine the orientation of the rectangular shapes (1 = horizontal; 2 = vertical).

#### Recognition task

Following the perception task, participants performed a recognition memory task for a sub-set of the pictures presented in the perception task. Of the total of 224 emotional (112) and neutral (112) pictures presented during the perception task, 160 (80 emotional and 80 neutral) were pseudo-randomly selected for the recognition memory task. Half of the 160 selected were Lo-Load and half were Hi-Load, and half were Short Dur and half Long Dur. This resulted in 20 emotional, Lo-Load, Short Dur; 20 emotional, Hi-Load, Short Dur; 20 emotional, Lo-Load, Long Dur; 20 emotional, Hi-Load, Long Dur; 20 neutral, Lo-Load, Short Dur; 20 neutral, Hi-Load, Short Dur; 20 neutral, Lo-Load, Long Dur; 20 neutral, Hi-Load, Long Dur. The 160 old images were pseudo-randomized with 80 new images selected from the same original picture databases and were selected on arousal and valence scores as well as similar semantic content. Averaged normative arousal and valence scores for Old and New emotional and neutral items, respectively, were as follows: 5.93/2.63 for Emotional old pictures; 5.95/2.66 for Emotional new pictures; 3.41/5.04, for Neutral old pictures; and 3.41/5.02 for Neutral new pictures. Arousal and valence scores were assessed using nine-point Likert scales, as follows: Arousal (1 = Lowest/9 = Highest), Valence (1 = Very Negative, 5 = Neutral, and 9 = Very Positive). Pairwise comparisons showed that emotional pictures had significantly greater arousal scores and lower valence scores than the neutral pictures, but there were no differences between the scores for emotional or neutral pictures from different categories.

### Experimental procedures

The 240 trials were divided into five runs of 48 trials (16 Emotional old, 16 Neutral old, 8 Emotional new, 8 Neutral new). Old stimuli were pseudo-randomized based on when they appeared in the perception task to ensure that a delay of approximately 40 min occurred between the encoding and retrieval of a stimulus. For example, if a picture was presented in the first run of the perception task, then it would be presented in either the first or second run of the recognition task. Likewise, if a stimulus was presented in the last run of the perception task then it was presented in the last run of the recognition task. To avoid induction of longer-lasting mood states, the trials within each run were pseudo-randomized, so that no more than two trials of the same valence type were consecutively presented. Each picture was displayed for 2000 ms during which the participant had to indicate with a button press whether it was an “*Old*” or a “*New*” image. Immediately following this 2000 ms response window a confidence rating screen appeared for 2000 ms asking the participant to rate the confidence of their decision on a three-point Likert scale (1 = lowest, 3 = highest). Each trial was followed by a jittered fixation interval drawn from an exponential distribution with a median of 6 s and a range from 4 to 12 s. Participants were not aware that a memory task would come following the perceptual task – they were told that the perception task would last for the entire time they were in the scanner. However, the perception task lasted approximately 55 min after which the experimenter instructed them that they would be performing a memory task for items that were presented in the perception task. The memory task did not begin until the participants confirmed that they understood the instructions for the task.

### Imaging protocol

Collection of MRI data was conducted on a 1.5-T Siemens Sonata scanner. After the sagittal localizer and the 3-D magnetization prepared rapid acquisition gradient-echo anatomical series [field of view (FOV) = 256 mm × 256 mm, repetition time (TR) = 1600 ms, echo time (TE) = 3.82 ms, number of slices = 112, voxel size = 1 mm^3^), a series of functional volumes allowing for full-brain coverage were acquired axially, using an echoplanar sequence (FOV = 256 mm × 256 mm, TR = 2000 ms, TE = 40 ms, number of slices = 28, voxel size = 4 mm × 4 mm × 4 mm, flip angle = 90°).

### Behavioral data analysis

The immediate impact of emotion on perception was measured as reaction time (RT) to making orientation (vertical vs. horizontal) discrimination decisions to the rectangular pictures. An initial analysis was performed similar to that from the report focusing on the immediate effect of emotional distraction (Shafer et al., 2012), and involved a repeated measures ANOVA with three within subjects variables [Emotion (Emo, Neu); Load (Lo, Hi); Duration (Short, Long)]. However, to establish the link between the immediate and long-term effects of emotion, the present focus was on items that were both correct in the perception task and also later remembered in the memory task (Hits), and involved data from subjects that had at least four trials per condition (11 subjects met this criterion). This analysis was done to ensure that similar behavioral effects existed for the perception task after reducing the number of subjects and trials per subject as only items from the perception task that were also in the memory task were assessed. The long-term impact of emotion on memory was assessed as corrected recognition scores [% Hits – % False Alarms (FA)], using repeated measures ANOVA with the same three variables. Corrected recognition scores were involved because their calculation is a common and stringent technique of assessing accuracy in memory tasks, as it considers responses to both *Old* (Hits and Misses) and *New*/foil (Correct Rejections and False Alarms) items. Even though confidence ratings were acquired during the recognition task, they were collapsed for analyses in order to increase statistical power.

**Figure 2:**
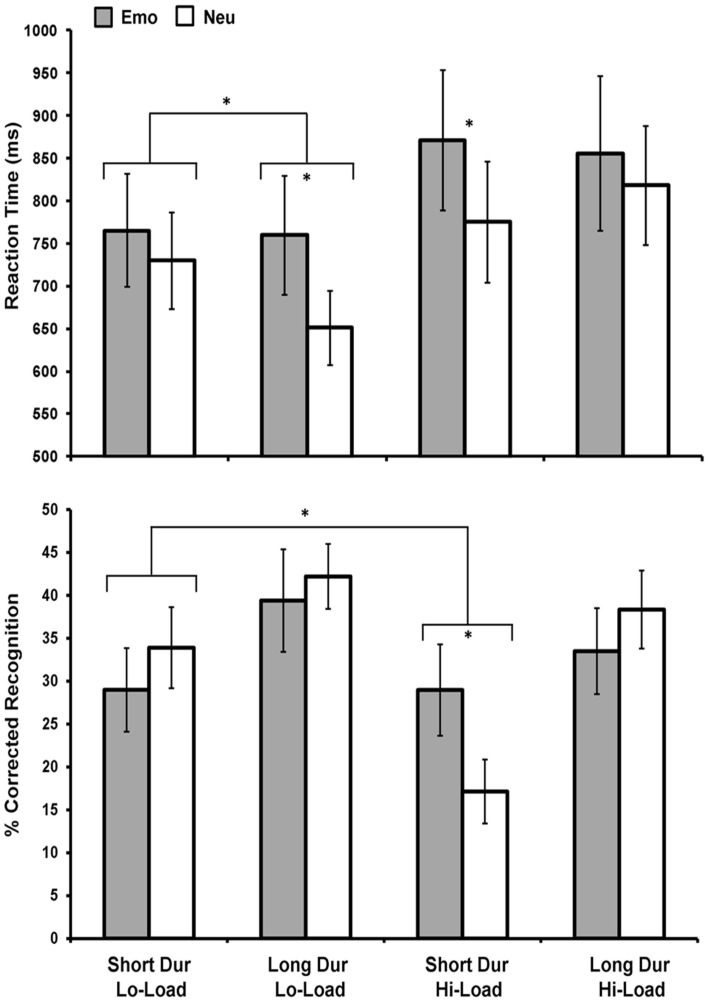
**Emotional distraction impaired perceptual performance under increased availability of processing resources while enhanced memory for task-irrelevant emotional distraction occurred only under limited processing resources**. Figure shows average reaction time (top panel) and corrected recognition data (bottom panel) for correctly identified rectangles during the perception task for 11 participants. Impaired perceptual task performance for Emo distracters was greatest when resources were most available (under conditions of Lo-Load and Long Dur). Instead, enhanced memory for Emo distracters was found only when resources were the most limited (for Hi-Load and Short Dur trials). An interaction was also found between emotion and load under short stimulus duration due to decreased memory for Neu distracters under conditions of limited resources, while memory for Emo distracters remained unaffected. Emo, Emotional; Neu, Neutral; Lo-Load, Low Perceptual Load; Hi-Load, High Perceptual Load; Dur, Stimulus Duration. *Significant at *p* ≤ 0.05, two-tailed.

**Figure 3:**
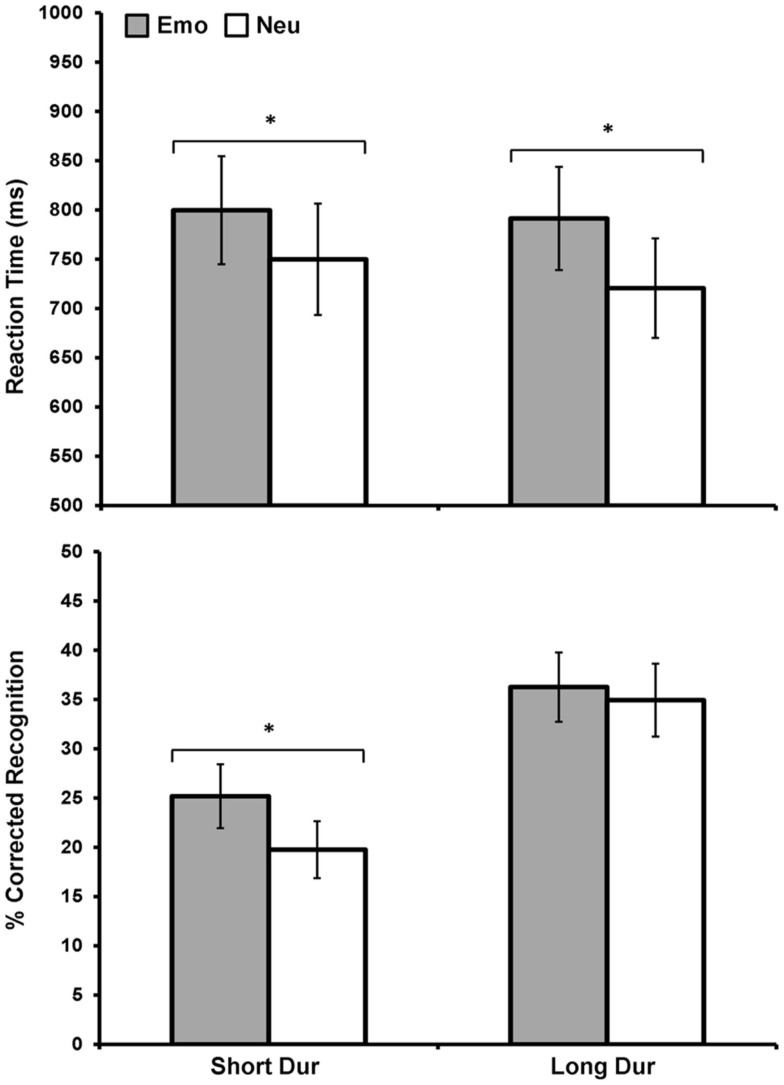
**Direct relationship between emotional distraction and memory, under limited resource availability**. Figure shows the item categories meeting both criteria – impaired perception and enhanced memory – for 16 subjects, after collapsing the Load variable. Top panel shows average response latency data for correctly identified rectangles that were later remembered. Bottom panel shows average corrected recognition data for the same items. As illustrated, the left side of the two graphs shows the direct relationship between emotional distraction and memory under limited resources during encoding, by identifying the items for which the opposing immediate/impairing vs. long-term/enhancing effects co-occur. Although these opposing effects are driven by the Hi-Load condition (as evident in Figure 2), it was necessary to collapse Load to increase power for analyses of brain imaging data using the subsequent memory paradigm. Emo, Emotional; Neu, Neutral; Dur, Stimulus Duration. *Significant at *p* ≤ 0.05, two-tailed.

Following these initial assessments on 11 subjects, to increase statistical power for both behavioral and fMRI analyses, data for the Load condition were collapsed together to maximize the possibility of comparing both the immediate and long-term effects of emotional distraction on perception and memory. In considering the main goal of the study (i.e., identification of common neural correlates of the opposing effects of emotional distraction), it was necessary to focus on conditions where the opposing effects of emotion were seen behaviorally, as this was the basis of our fMRI investigation. These opposing effects were identified in only one condition (i.e., Short Dur Hi-Load – see the third set of bars from left in the top and bottom panels of Figure 2). While, ideally, would have been to investigate the neural correlates of these opposing effects in the Hi-Load condition only, separation according to all conditions was possible only in data from 11 subjects. Hence, to increase the statistical power for brain imaging analyses, it was necessary to collapse the Load condition. This was the most valid choice for further analyses, as collapsing Load maintained the opposing effects (see first set of bars in Figure 3), and thus allowed us to perform the fMRI analyses corresponding to these behavioral effects on data from 16 subjects. Although collapsing Load might have overall weakened the effects observed in the fMRI data, seemingly driven by the Hi-Load condition (compare Figures 2 and 3), this was a necessary and advantageous trade-off, as it allowed for investigation of data from more subjects, although our sample size in this follow-up investigation (*N* = 16) was slightly smaller than what is suggested for the use of brain-behavior relationships (Lieberman et al., 2009), which we employed in the original report (*N* = 18; Shafer et al., 2012). Furthermore, collapsing Load conditions was critical, as also described below, to identify brain activity associated with the impact of emotion on memory using the subsequent memory paradigm (Dolcos et al., 2004b, 2011; Shafer et al., 2011), because it allowed analysis of data when considering Emotion (Emo vs. Neu), Duration (Short vs. Long), and Memory (Remembered vs. Forgotten) variables.

Again, when analyzing data from the larger sample (*N* = 16), to establish the link between the immediate and long-term effects of emotion in the behavioral data, the immediate impact of emotion was calculated on the items that were also later remembered in the memory task (Hits). The immediate and long-term effects of emotion were examined by performing a repeated measures ANOVA [Emotion (Emo, Neu); Duration (Short, Long)] on RT and corrected recognition data, respectively. Importantly, these analyses allowed us to examine how manipulations of attentional demand at encoding for task-irrelevant emotional items influenced emotion’s long-term impact on memory. Pairwise comparisons were Bonferroni corrected.

### fMRI data analysis

Imaging data analyses were performed on data from 16 participants, using SPM in conjunction with in-house custom Matlab scripts. Statistical analyses were preceded by the following preprocessing steps: quality assurance, TR alignment, motion correction, coregistration, normalization, and smoothing (8 mm^3^ Kernel). For individual analyses, task-related activity was identified by convolving a vector of the onset times of the stimuli with a synthetic hemodynamic response and its temporal derivative. The general linear model, as implemented in SPM2, was used to model the effects of interests and other confounding effects (e.g., session effects and magnetic field drift). There were 14 first-level regressors: eight task variables (Emo Long Dur Hits, Emo Short Dur Hits, Neu Long Dur Hits, Neu Short Dur Hits, Emo Long Dur Misses, Emo Short Dur Misses, Neu Long Dur Misses, Neu Short Dur Misses) +6 motion regressors (three translations, three rotations). Group analyses were conducted using random-effects models to assess the effect of distracter content and stimulus duration on perception and memory processes. Based on the behavioral results and to increase statistical power, as mentioned above, the analyses of fMRI data assessed emotion’s interaction with stimulus duration (Short vs. Long), which yielded the strongest effects of emotion on both perception and memory. Furthermore, to ensure that subjects had maintained focus on the primary task and also in accordance with the behavioral data where Hits were driving the main effect of emotion on perceptual performance, the fMRI data analyses for the immediate effect of emotion were performed on items presented during the perception task that were performed correctly in the perception task and that were later remembered (Hits). For the analyses of the long-term impact, subsequent memory effects were calculated for emotional and neutral items and then compared to each other (Dolcos et al., 2004b, 2011; Shafer et al., 2011). As with the analyses concerning the immediate effect of emotion, fMRI data analyses for the long-term effect only included items were correct in the perception task.

The main goal of fMRI data analyses was to identify the neural correlates linking the immediate impact of emotional distraction on perception and the long-term impact of emotion on memory, and to identify the neural correlates specific to one or the other of these effects. To accomplish this goal, we compared activity in brain regions specifically sensitive to the presence of emotional distraction and activity in brain regions sensitive to the emotional enhancement of memory. First, paralleling the behavioral data, we investigated areas associated with emotional distraction for the short duration condition. A *t*-map was computed contrasting short emotional (Emo Short Dur Hits) vs. short neutral (Neu Short Dur Hits) items.

Next, we investigated areas associated with the emotional enhancement of memory. Areas of brain activity reflecting the emotional enhancement of memory during encoding found for the short duration condition in the behavioral data were examined by employing subtraction analysis looking at differences in activity between remembered (Hits) and forgotten (Misses) items (Dm/Subsequent Memory Effect) for Emo Short Dur compared to Neu Short Dur stimuli. First we computed *t*-maps for differences in activity due to memory for Emo and Neu Short Dur items separately [Emo Short Dur Dm = (Emo Short Dur Hits−Emo Short Dur Misses), Neu Short Dur Dm = (Neu Short Dur Hits−Neu Short Dur Misses)]. Then, to identify activity associated with the emotional enhancement of memory, we employed subtraction analysis where the individual *t*-map for Neu Short Dur Dm was subtracted from the individual *t*-map for Emo Short Dur Dm. To make sure that these differences were based on an existing Dm effect for the emotion condition and were not driven by negative Dm for the neutral condition, this interaction was then inclusively masked by Emo Short Dur Dm [(Emo Short Dur Dm−Neu Short Dur Dm) ∩ (Emo Short Dur Dm)]. Lastly, to ensure that activity was unique to the behavioral effects found in the Short Dm condition, we exclusively masked the above resulting contrast with activity that was present when assessing emotional memory for the long duration condition [(Emo Long Dur Dm−Neu Long Dur Dm) ∩ (Emo Long Dur Dm)]. As with the behavioral data, we collapsed confidence ratings in the fMRI analyses in order to increase statistical power. While this prevented us from disentangling similarities and differences between emotional distraction on recollection vs. familiarity memory processes, by separately examining high vs. low confidence responses (Daselaar et al., 2006; Hayes et al., 2011), we did find the majority of responses to be high in confidence and therefore our data may be more indicative of recollection processes (confidence ratings distribution: high = 71%, medium = 20%, low = 9%).

After separately identifying the neural correlates of the immediate and long-term effects of emotion, we investigated brain regions that contribute both to emotion’s initial impact on perception and attention and to emotion’s enhancement of memory. To identify brain regions responsible for both effects, we examined overlapping areas of activation between the immediate and long-term impact of emotion using a conjunction analysis. This was performed using the contrast for the effect of emotion during perception for the Short Dur condition and the contrast for the emotional enhancement of memory during the Short Dur condition (Emo Short Dur Hits > Neu Short Dur Hits) ∩ {[(Emo Short Dur Dm − Neu Short Dur Dm) ∩ (Emo Short Dur Dm)], exclusively masked by [Emo Long Dur Dm − Neu Long Dur Dm) ∩ (Emo Long Dur Dm)]}.

Finally, to dissociate areas that showed specificity only to immediate or long-term effects of emotion, we exclusively masked the contrasts computed above. For example, to identify activity associated only with the long-term effect of emotion, we exclusive masked the contrast associated with the long-term effect with that of the immediate effect and vice-versa when identifying activity unique to the immediate effect. Also, to investigate the significance of overlapping or dissociating activations, brain-behavioral relationships were investigated by correlating brain activity with indices of performance (RTs for the immediate and Corrected Recognition scores for the long-term effects). These latter analyses targeted MTL emotion (AMY) and memory (HC) structures.

Cortical structures were assessed with a threshold of *p* ≤ 0.005, uncorrected, and *a priori* MTL areas of interest were assessed with a threshold of *p* ≤ 0.05; in addition, for all interaction analyses an intensity threshold of *p* ≤ 0.05 was employed. These thresholds were selected to stay consistent with our previous report using the same task (Shafer et al., 2012), so that similar inferences could be made across reports. It should also be noted that the interactions were masked by specific main effects using an intensity threshold of *p* ≤ 0.005. Hence, the joint probability of the resulting conjunction maps was of *p* ≤ 0.00025, which is the product of their independent probabilities (0.05 × 0.005; Fisher, 1950). Similarly, for all interaction analyses examining MTL regions (i.e., AMY and HC) an intensity threshold of *p* ≤ 0.05 was employed for the interaction, which was then masked by a specific effect using an intensity threshold of *p* ≤ 0.05. Hence, the joint probability of the resulting conjunction map was of *p* ≤ 0.0025. Finally, for correlation analyses in MTL emotion- and memory-related regions a threshold of *p* ≤ 0.05 was used and all correlation maps were also masked by the statistical map that they were being correlated with. For example, in MTL regions for the immediate effect of emotion, a double conjunction was used where the correlation map (*p* ≤ 0.05) was inclusively masked by the effect of emotion for the Short Dur condition (*p* ≤ 0.05), resulting in a joint probability of *p* ≤ 0.0025. Similarly, for the long-term effect of emotion a triple conjunction was used *p* ≤ 0.05 for the correlation map, *p* ≤ 0.05 for the interaction, and *p* ≤ 0.05 for the Emo Short Dm, thus the resulting probability was *p* ≤ 0.000125. Details about the joint thresholds are provided in the legend of each figure and table. An extent threshold of five contiguous voxels was used in all analyses.

## Results

### Behavioral results

#### Direct relationship between immediate and long-term impact of emotional distraction, in the context of overall dissociating impairing vs. enhancing effects

Unlike the immediate impact of emotional distraction on perceptual processing, which was greatest when processing resources were most available (easy task and long presentation time), the long-term impact of emotion on memory was the strongest when processing resources were least available (difficult task and short presentation time). Initial analysis (*n* = 11) on RT data for the immediate impairing effect of emotional distraction on perception showed a main effect of Emotion, *F*(1, 10) = 10, *p* = 0.01, Load, *F*(1, 10) = 8.03, *p* = 0.02, and an Emotion × Load × Duration interaction, *F*(1, 10) = 5.34, *p* = 0.04. As previously found with a larger sample (Shafer et al., 2012), trials with negative distracters took longer to respond to than those with neutral distracters and Hi-Load trials took longer to respond to than Lo-Load trials. Furthermore, the three-way interaction was driven by an Emotion × Duration when Load was low, *F*(1, 10) = 5.59, *p* = 0.04, but not high, *F*(1, 10) = 0.629, *p* = 0.45 (see Figure 2, top panel). Analysis on corrected recognition data (*n* = 11) revealed a main effect of Load, *F*(1, 10) = 5.39, *p* = 0.04, and Duration, *F*(1, 10) = 23.34, *p* ≤ 0.001, but no main effect of Emotion. However, a marginally significant Emotion × Load × Duration interaction was present, *F*(1, 10) = 3.82, *p* = 0.08, and *post hoc* analyses showed that this interaction was driven by an Emotion × Load interaction for short duration items, *F*(1, 10) = 7.84, *p* = 0.02. Specifically, emotion significantly affected memory in the Hi-Load, *t*(10) = 2.31, *p* = 0.04, but not in the Lo-Load, *t*(10) = 0.976, *p* = 0.35, condition for Short Dur items (see Figure 2, bottom panel).

As mentioned in Section “Materials and Methods,” to increase statistical power for both behavioral and fMRI analyses, data for the Load condition were collapsed together to maximize the possibility of comparing both the immediate and long-term effects of emotional distraction on perception and memory, respectively. This was critical to identify brain activity associated with the impact of emotion on memory using the subsequent memory paradigm (Dolcos et al., 2004b, 2011; Shafer et al., 2011). Collapsing load allowed us to include 16 subjects in our behavioral and imaging analysis for the memory data.

Importantly, collapsing load allowed for identification with increased statistical power of common effects for the immediate (impairing) and long-term (enhancing) impact of emotion, which occurred for the short presentation time (250 ms; see Figure 3). Emotional distracters that were later remembered had a significant effect on discrimination performance such that there was delayed RT when distracters were emotional compared to neutral, *F*(1, 15) = 9.99, *p* = 0.006. This effect of emotion was found for both short, *t*(15) = −2.15, *p* = 0.05, and long, *t*(15) = −2.56, *p* = 0.02, duration conditions (Figure 3, top panel). Examination of corrected recognition scores also with load conditions collapsed together, for the items that were presented previously as distracters during the perception task, revealed an effect of emotion only for the short condition *t*(15) = 2.1, *p* = 0.05 (Figure 3, bottom panel). Analyses also identified a significant main effect of duration, *F*(1, 15) = 26.06, *p* ≤ 0.001, with memory performance being overall better for long vs. short duration items.

In summary, the behavioral data showed that the long-term impact of emotion on memory was the strongest when processing resources were least available, and both the immediate and long-term effects of emotion (albeit opposing) occurred for the short duration items. Hence, the fMRI analyses focused on identifying common and dissociable neural correlates associated with those items.

### fMRI results

#### Common brain regions for the immediate and long-term impact of emotion

**Figure 4:**
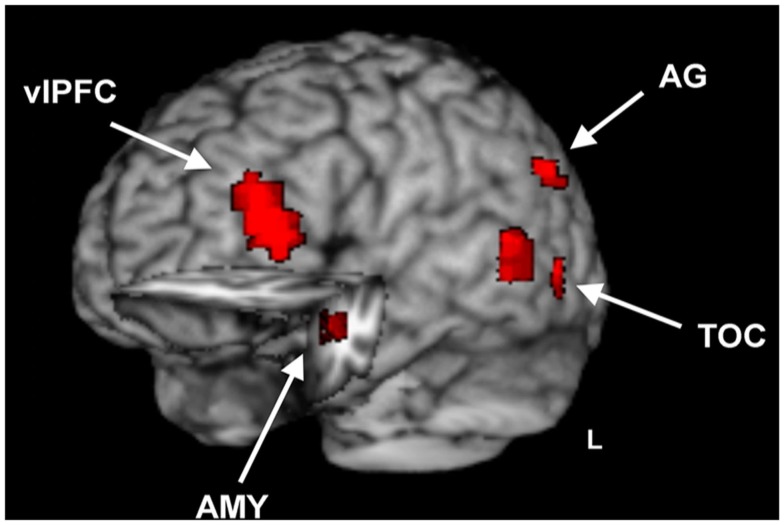
**Brain regions sensitive to opposing emotional modulation during emotional distraction vs. memory**. Image shows common regions of response to the impairing effect of emotional distraction and the enhancing effect of emotional memory, superimposed on a high resolution brain image displayed in a tridimensional view using MRIcron (http://www.mccauslandcenter.sc.edu/mricro/mricron/). Cut-out in the left hemisphere also reveals similar response to emotional distraction and memory in the left AMY and anterior HC. The conjunction activation maps contributing to the overlap were identified separately for immediate/distraction and long-term/enhanced memory for the short Dur condition. The maps contributing to the final conjunction map were separately created for the immediate and long-term impact, which each resulted from maps with *p* < 0.005, *p* < 0.05, *p* < 0.005 for the main effect of emotion, interaction, and mask maps, respectively, for areas outside of the MTL, and *p* < 0.05, *p* < 0.05, and *p* < 0.05 for the main effect of emotion, interaction, and mask maps, respectively, for areas within the MTL (AMY, HC); see Section “Materials and Methods” for details. vlPFC, ventrolateral Prefrontal Cortex; TOC, Temporal Occipital Cortex; AG, Angular Gyrus; AMY, Amygdala; HC, Hippocampus; L, Left.

Investigation of overlapping effects of emotion on perception and memory in the Short Dur condition identified common areas of activation in ventrolateral PFC (vlPFC), temporal occipital cortex, in the left angular gyrus (AG), precuneus, and left AMY and hippocampus (HC; see Figure 4 and Table 1).

**Table 1:** **Common areas of activation for the immediate and long-term effects of emotion**.

Brain regions		Talairach coordinates				*T* values		Cluster size
		BA	*x*	*y*	*z*	Immediate	Long term interaction/mask	
lPFC	R. Middle frontal gyrus	9	43	18	28	4.2	2.64/3.17	20
vlPFC	L. Inferior frontal gyrus	45	−45	28	13	5.08	5.6/3.63	49
	R. Inferior frontal gyrus	45	47	20	14	4.56	3.44/3.04	20
PoCG	R. Post central gyrus	43	54	−11	15	3.47	3.28/3.38	6
PC	L. Angular gyrus	39	−50	−68	29	4.45	4.65/4.62	6
	L. Precuneus	7	−2	−57	35	3.68	3.23/3.47	7
TOC	l. Middle temporal gyrus	21	−57	−47	6	4.02	3.28/3.68	15
	R. Middle temporal gyrus	19	39	−75	19	4.29	3.09/3.19	19
	L. Inferior temporal gyrus	37	−46	−65	1	4.15	1.91/3.53	5
	R. Inferior temporal gyrus	19	40	−65	−5	4.32	2.78/3.91	26
	L. Fusiform Gyrus	37	−42	−50	−5	4.75	2.52/3.14	12
	R. Fusiform Gyrus	37	36	−49	−14	3.78	2.06/3.46	14
	R. Superior occipital gyrus	19	35	−76	27	3.53	2.77/3.31	19
MTL	L. Amygdala		−27	−4	−15	3.05	2.49/1.82	21
	L. Hippocampus		−27	−39	−3	3.5	3.13/2.56	18
	L. Uncus	28	−23	8	−21	4.08	2.65/2.34	32
	L. Parahippocampus	34	−27	4	−14	4.5	3.37/1.87	32
		36	−34	−34	−14	3.65	2.32/2.41	18
Midbrain	L. Substania nigra		−8	−12	−11	4.08	3.69/3.28	8

*Table identifies brain regions associated with the both the immediate (impairing) and long-term (enhancing) effects of emotion. Regions were identified by a conjunction map between separately identified regions for immediate and long-term effects. To make sure differences for the long-term effect of emotion were based on an existing Dm effect for the emotion condition and were not driven by negative Dm for the neutral condition, the long-term interaction was inclusively masked by Emo Short Dur Dm = (Emo Short Hits > Emo Short Misses). To ensure activity for the long-term effect of emotion was associated only with the behavioral effect seen for the Short Dur condition, the *t*-map for long-term effect of emotion for Short Dur was exclusively masked by the long-term effect of emotion for the Long Dur. Conjunction map = Immediate map (Emo Short Dur Hits > Neu Short Dur Hits) ∩ Long-term map {[(Emo Short Dm) vs. (Neu Short Dm) ∩ (Emo Short Dm)], exclusively masked by [(Emo Long Dm) vs. (Neu Long Dm) ∩ (Emo Long Dm)]}. lPFC, lateral Prefrontal Cortex; vlPFC, Ventral Lateral Prefrontal Cortex; PoCG, Post Central Gyrus; PC, Parietal Cortex; TOC, Temporal Occipital Cortex; MTL, Medial Temporal Lobe. *T* values reported for cortical regions met the criteria of *p* < 0.005, *p* < 0.05, and *p* < 0.005, for the immediate effect, long-term interaction and long-term mask, respectively; values reported for the MTL regions met the criteria of *p* < 0.05 for all effects*.

#### Hemispheric disassociation in the amygdala and hippocampus linked to emotional distraction and memory

**Figure 5:**
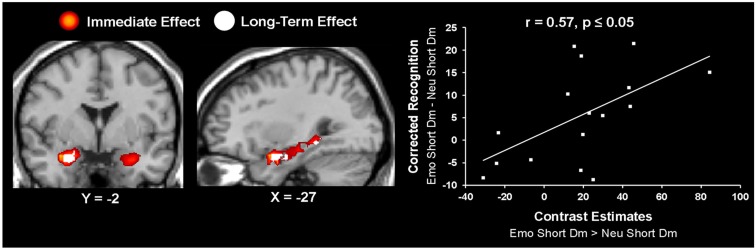
**Hemispheric dissociation linked the immediate vs. long-term effects of emotion in the amygdala and hippocampus**. Left panel shows a coronal view of the AMY, highlighting the lateralization effect showing the bilateral immediate effect of emotion (in red) and the left-lateralized long-term effect of emotion (in white). The middle panel shows a sagittal view of the three left hemisphere AMY-HC areas identified for the long-term effect of emotion. These activations are overlapped on the activity identified for the immediate effect of emotion. The right panel shows a scatterplot illustrating the results from the peak voxel of the correlation calculated on the contrast estimates from the long-term effect of emotion during the short Dur condition and the corresponding behavioral data, as extracted from left AMY (Talairach coordinates: *x* = −27, *y* = −4, *z* = −15). The contrast used for creating the correlation *t*-maps was [(Emo Short Dm vs. Neu Short Dm) ∩ (Emo Short Dm)]. The resulting joint probability for the correlation *t*-map is *p* < 0.000125; see Section “Materials and Methods” for details. Emo, Emotional; Neu, Neutral; Dur, Stimulus Duration; Dm, Difference due to memory; AMY, Amygdala; HC, Hippocampus.

In addition to identifying brain regions associated with both the immediate and long-term effects of emotion on perception and memory, areas that dissociated between these effects were also identified. This analysis identified a hemispheric disassociation in the AMY and HC, which although showed bilateral activation in response to emotional distraction, showed memory-related activity only in the left hemisphere (see Figure 5). To further explore whether this disassociation was indicative of functional asymmetry, we extracted functional regions of interest (ROI) for the three clusters of activity identified in these regions for the long-term effect (i.e., left AMY, anterior HC, and posterior HC) and their homologous counterparts in the right hemisphere. Each functional ROI was comprised of the peak voxel of each cluster along with its neighboring voxels. We then conducted a repeated measures ANOVA with Emotion and Hemisphere as within subject variables for each of the three clusters. Results for the AMY and anterior HC clusters were similar and showed a main effect of Emotion [AMY, *F*(1, 15) = 6.39, *p* = 0.02; anterior HC, *F*(1,15) = 8.39, *p* = 0.01], but no effect of Hemisphere or interaction between Emotion and Hemisphere. However, the posterior HC cluster, not only showed a main effect of Emotion, *F*(1, 15) = 5.63, *p* = 0.03, but also an Emotion × Hemisphere interaction, *F*(1, 15) = 7.75, *p* = 0.02). *Post hoc* analysis revealed that differences between emotional and neutral short Dm were significant in the left, *t*(15) = 3.96, *p* = 0.001, but not right hemisphere *t*(15) = 0.18, *p* = 0.86. While the Emotion × Hemisphere interaction in the AMY and anterior HC clusters was not significant, *post hoc* examination showed the left hemisphere to indeed have stronger statistical difference between emotional and neutral short Dm compared to the right hemisphere; L. AMY, *t*(15) = 2.87, *p* = 0.01; R. AMY, *t*(15) = 1.89, *p* = 0.08; L. anterior HC, *t*(15) = 2.81, *p* = 0.01; R. anterior HC, *t*(15) = 2.01, *p* = 0.06.

Further investigation of activity in these regions using brain-behavior correlations revealed that the left AMY activity identified for the long-term effect of emotion on memory was correlated with the corresponding behavioral difference in memory performance, *r* = 0.57 *p* ≤ 0.05 (Figure 5); activity in the left anterior HC (Talairach coordinates: *x* = −30, *y* = −7, *z* = −15) also correlated with memory performance, *r* = 0.55, *p* ≤ 0.05, but the cluster size was less than five voxels. In addition, a positive brain-behavior co-variation was also identified between activity in the left entorhinal cortex (Talairach coordinates: *x* = −16, *y* = 4, *y* = −17) and RT during the perceptual task, but this effect was not specific to emotional distraction (*r* = 0.7, *p* ≤ 0.05), as the same relationship was found for the neutral items (*r* = 0.69, *p* ≤ 0.05).

#### Emotional distraction vs. memory-specific brain activity

Analysis investigating specific response to the immediate vs. long-term impact also identified activity linked only to the immediate impact of emotional distraction. This analysis identified a number of brain areas to have regional or sub-regional specificity, with areas being only involved in the immediate impact of emotional distraction or contributing to both immediate and long-term effects, respectively. Sub-regional specificity was found in the superior frontal gyrus, AG, inferior frontal gyrus, post central gyrus, precuneus, cingulate gyrus, fusiform gyrus, inferior and middle temporal gyri, as well as left AMY, HC, and paraHC regions. For example, inferior frontal gyrus (Brodmann Area 45) was identified for the immediate and long-term impact, whereas Brodmann Area 47 was associated with only the immediate impact of emotional distraction on perception. Regions that exhibited specificity to the immediate effect of emotional distraction included, medial frontal gyrus, precentral gyrus, superior temporal gyrus, and middle occipital gyrus (see Tables 1 and 2).

**Table 2:** **Dissociable areas of activation for the immediate and long-term effects of emotion**.

Brain regions		Talairach coordinates				*T* values		Cluster Size
		BA	*x*	*y*	*z*	Immediate	Long-term interaction/mask	
**IMMEDIATE**								
mPFC	L. Superior frontal gyrus	8	−9	39	47	6.66		83
	R. Superior frontal gyrus	8	6	27	54	4.66		
	L. Medial frontal gyrus	10	−5	51	5	6.31		6
lPFC	L. Middle frontal gyrus	6/8	−39	10	44	3.95		13
vlPFC	L. Inferior frontal gyrus	47	−41	26	−8	4.12		6
		46	−42	40	4	5.6		29
	R. Inferior frontal gyrus	47	40	36	−2	3.96		43
Insula	L. Insula	13	−34	8	−14	5.30		83
PrCG	R. Precentral gyrus	4	50	−6	44	3.89		7
PoCG	R. Postcentral gyrus	3	28	−33	49	3.33		5
Cingulate	L. Cingulate gyrus	31	−13	−25	45	4.86		20
PC	L. Inferior parietal lobe	40	−54	−39	43	3.6		7
	L. Precuneus	7	−35	−73	44	4.55		75
	R. Precuneus	7	17	−73	44	3.59		8
	L. Angular gyrus	39	−53	−64	30	4		75
	R. Angular gyrus	39	39	−76	30	3.51		5
TOC	L. Fusiform gyrus	37	−49	−45	−12	4.80		80
		20	−34	−37	−18	4.72		
	L. Middle temporal gyrus	21	−49	9	−28	3.51		7
		37	−49	−66	4	4.65		47
	L. Inferior temporal gyrus	20	−49	−7	−19	3.76		13
	L. Superior temporal Gyrus	22	−64	−36	7	3.93		16
	R. Middle occipital gyrus	18	28	−92	3	4.03		7
MTL	L. Amygdala		−19	−4	−14	2.85		30
	R. Amygdala		21	0	−17	3.21		41
	L. Hippocampus		−27	−19	−12	3.59		76
	R. Hippocampus		29	−23	−12	2.88		65
	R. Parahippocampus	28	25	−23	−12	3.72		8
		30	14	−35	−2	3.21		10
		34	25	4	−17	3.59		5
Subcortical	R. Thalamus-Pulvinar		17	−32	5	3.91		10
Midbrain	R. Substania nigra		10	−23	−9	3.55		9
	R. Red nucleus		6	−16	8	3.74		
Cerebellum	L. Culmen		−8	−46	−7	3.47		10
**LONG-TERM**								
mPFC	L. Superior frontal gyrus	9	−5	56	24		2.85/4.13	6
Insula	R. Posterior insula		32	−29	13		2.33/3.37	5
Cingulate	L. Anterior cingulate	32	−1	30	22		3.83/3.2	5
		24	−8	43	12		3.06/3.9	6
	R. Anterior cingulate	32	3	42	16		2.9/3.03	6
PC	R. Precuneus	7	5	−74	55		3.17/3.25	8
	R. Superior parietal lobe	7	24	−65	35		3.04/3.32	7
	L. Superior parietal lobe	7	−17	−59	52		2.94/3.64	5
Subcortical	L. Putamen		−23	1	11		3.22/3.58	7

*Table identifies brain regions associated with the either the immediate or long-term effect of emotion. Immediate effect = (Emo Short Dur Hits > Neu Short Dur Hits), exclusively masked by the long-term effect of emotion at *p* < 0.05. The long-term effect of emotion was found by calculating the interaction between Emo Short Dm vs. Neu Short Dm. This interaction was then inclusively masked by Emo Short Dur Dm, to make sure the differences were based on an existing Dm effect for the emotion condition and were not driven by negative Dm for the neutral condition. To ensure activity for the long-term effect of emotion was associated only with the behavioral effect seen for the Short Dur condition, the *t*-map for long-term effect of emotion for Short Dur was exclusively masked by the long-term effect of emotion for the Long Dur. Long-term effect = {[(Emo Short Dm) vs. (Neu Short Dm) ∩ (Emo Short Dm)], exclusively masked by [(Emo Long Dm) vs. (Neu Long Dm) ∩ (Emo Long Dm)]}. Lastly, the entire long-term effect was exclusively masked by the immediate effect of emotion at *p* < 0.05. mPFC, Medial Prefrontal Cortex; lPFC, lateral Prefrontal Cortex; vlPFC, Ventral Lateral Prefrontal Cortex; PrCG, Precentral Gyrus; PoCG, Post Central Gyrus; PC, Parietal Cortex; TOC, Temporal Occipital Cortex; MTL, Medial Temporal Lobe. Significance threshold for the immediate effect of emotion is *p* < 0.005 and *p* < 0.05, for cortical and MTL regions, respectively. Significance threshold for the long-term effect of emotion is *p* < 0.005 for the mask for cortical regions and *p* < 0.05 for targeted MTL regions and *p* < 0.05 for the interaction for both cortical and MTL regions*.

Analyses investigating specific response to the immediate vs. long-term impact also identified activity linked only to the memory-enhancing effect of emotion. Again, as with the immediate impact reported above, sub-regional and regional specificity were found. Sub-regional specificity was identified in the superior frontal gyrus, cingulate gyrus, and precuneus. Of the activity identified as being unique to the long-term impact of emotion on memory only one region, the superior parietal lobe was solely specific to emotional memory.

Collectively, the analyses of fMRI data targeting activity associated with the conditions that had opposing effects of emotional distraction on immediate and long-term processing identified both areas of overlap and areas dissociating these two effects. The overlapping areas are involved in the mechanisms responsible for both the immediate/impairing impact of emotional distraction on perception and for the long-term/enhancing impact on memory for the distracters themselves. Areas dissociating between these two effects were found to do so with either regional or sub-regional specificity. These findings will be discussed in detail below.

## Discussion

The present study used an experimental paradigm that manipulated the degree of resource availability for processing task-irrelevant emotional distraction, to determine how the initial impact of emotional distraction is related to the long-term impact on memory for the distracters themselves. Our study yielded three main findings. First, we observed a direct relationship between the immediate (impairing) and long-term (enhancing) impact of emotion, only under conditions of limited resources during encoding. Second, linked to this behavioral effect, we identified a number of brain regions of the emotion network that were involved in both the immediate and long-term impact of emotion, including AMY-HC regions, the ventrolateral prefrontal, temporal occipital, and inferior parietal cortices. Third, responses in specific regions and sub-regions differentiated between immediate and long-term effects of emotion, both in terms of overall activation and co-variation with performance. Medial frontal, precentral, superior temporal, and middle occipital gyri activity was specifically associated with the immediate impact of emotion, whereas activity in superior parietal cortex was specifically associated with the long-term impact of emotion on memory. Furthermore, left AMY co-variation with subsequent memory performance and a hemispheric asymmetry of posterior HC activity in contributing to subsequent memory performance suggest a disassociation in the hemispheric contribution of these regions to the impact of emotional distraction on perception and memory.

### Direct relationship between immediate and long-term impact of emotional distraction, in the context of overall dissociating impairing vs. enhancing effects

The fact that a direct relationship between the immediate (impairing) and long-term (enhancing) impact of emotion only occurred under conditions of limited processing resources during encoding suggest that the immediate impact of emotional distraction does not translate into long-term effects in a one-to-one fashion. Thus, the conditions in which emotional distraction produces the strongest initial impact on perception do not necessarily lead to the strongest long-term impressions on memory for the distracters themselves. In other words, the aspects that we may remember most may not necessarily be those that initially distracted us while trying to perform a perceptual task. Instead, emotional distraction also produced a boost in long-term memory only under conditions of limited processing resources, which as also discussed below suggest that the direct link between opposing immediate (impairing) and long-term (enhancing) effects of emotional distraction under these circumstances involves automatic mechanisms. The engagement of such mechanisms to process task-irrelevant emotional information presented concurrently with a perceptual task led to reallocation of processing resources by emotional distraction, which in turn initiated processing that also resulted in better memory for the distracters themselves.

The absence of a direct link between the two opposing effects when more processing resources are available does not exclude the possibility that automatic mechanisms of emotion processing are also involved in circumstances that do not lead to a long-term memory advantage for emotional distraction. It is possible that, when more resources are available for processing during encoding, there is more opportunity for the mediated mechanisms to come “online” and influence memory for both emotional and neutral items, and hence the benefit that both emotional and neutral information receives from the mediated influences overshadows the memory boost produced for the emotional information by the automatic mechanisms alone. As a result, emotion’s impact on memory is diminished, although overall the memory performance is enhanced in conditions of increased engagement of mediated mechanisms at encoding (e.g., longer processing time). Although the effect of stimulus duration on memory is consistent with findings from research investigating the role of stimulus durations around this range (i.e., 250–1000 ms) on memory performance (Hulme and Merikle, 1976; Christianson and Fallman, 1990; Clark-Foos and Marsh, 2008), the absence of an emotion advantage is inconsistent with previous findings identifying such an effect within divided attention paradigms (Kern et al., 2005; Talmi et al., 2007; Pottage and Schaefer, 2012); it is consistent, however, with previous studies using level of processing paradigms where memory for neutral items may be on par with that of emotional items for deep levels of processing (Reber et al., 1994; Jay et al., 2008).

Elimination of the memory advantage for the emotional stimuli encoded in conditions of enhanced contribution of the mediated mechanisms may be due to a similar boost in memory performance for the neutral items or due to the engagement of mechanisms that diminished the impact of emotion on memory. Regarding the first possibility discussed earlier, with more resources available for distraction it is possible that the addition of mediated processes may have also benefited the neutral items, for instance, due to the engagement of working memory, semantic processing, and attentional processing (Dolcos et al., 2011). Regarding the alternative possibility, given our experimental design in which emotional information was task-irrelevant and participants were instructed to focus on the main perception task, it may be the case that under long stimulus duration, participants engaged processing to diminish the impact of emotional distraction. Thus, while they could not avoid being initially distracted by them (as indicated by the RT data in the perception task), trying to diminish their initial impact might have interfered with the mechanisms necessary for the emotional boost in memory performance. Importantly, however, we did observe a one-to-one relationship when only limited resources were available during the initial processing of emotional distraction.

### Common and dissociable brain regions for the immediate and long-term impact of emotion

Turning to the neural correlates of the link between the initial and long-term effects of emotional distraction on perception and memory, analyses of fMRI data identified a number of brain regions of the emotion processing network whose activity was linked to both the immediate/impairing and long-term/enhancing impact of emotion. Consistent with the engagement of automatic mechanisms linking the two opposing effects, we identified overlapping activity in AMY-HC regions, which have been linked to both emotion perception (Sergerie et al., 2008) and emotional memory (Dolcos et al., 2004b).

#### Hemispheric disassociation in the amygdala and hippocampus linked to emotional distraction and memory

Even though the functional ROI analysis did not confirm our impression of a hemisphere effect in the left AMY and anterior HC for the memory-enhancing effect of emotion, it did identify a hemisphere effect in the posterior HC. The general increased Emo Dm in the AMY and anterior HC is consistent with previous research (Dolcos et al., 2004b), and although there was no hemisphere effect in these two regions for the long-term effect, the increased statistical strength due to decreased variance in Emo Dm response observed in the left hemisphere comparisons, along with the left AMY brain-behavior co-variation, suggest a more consistent left hemisphere involvement in emotional memory for this task. This is consistent with findings from several studies of emotional memory (Canli et al., 2000; Mickley and Kensinger, 2008; Talmi et al., 2008; Mickley Steinmetz and Kensinger, 2009), although it is not consistent with findings of recent meta-analyses (Murty et al., 2010; Kim, 2011), which did not identify patterns of lateralization in the AMY linked to memory. One possibility is that in conditions of processing emotional information as task-irrelevant distraction the right AMY engages rapidly, producing a phasic response to the global arousal properties of the stimulus, thus extracting only crude information to prepare for immediate action. On the other hand, the engagement of the left AMY is associated with a tonic response reflecting the extraction of more specific information and elaborative processing of the emotional qualities of the stimuli, which also contributes to enhanced memory (Markowitsch, 1998; Phelps et al., 2001; Glascher and Adolphs, 2003; Sergerie et al., 2008). Furthermore, and as suggested by the increased variance in the right AMY and anterior HC for Emo Dm, the lack of right hemisphere involvement in emotional memory in these regions might be due to increased susceptibility of their right hemisphere response to individual differences.

#### Emotional distraction and memory-specific brain activity: increased medial frontal, precentral, superior temporal, and middle occipital activation linked to enhanced emotional distraction and increased parietal activation linked to enhanced emotional memory

Brain regions found to have specificity in response to emotional distraction or memory dissociate between areas that are susceptible to immediate emotional modulation from those that are susceptible to long-term emotional modulation. Importantly, these regions identify unique relationships that are specific to different points along the information processing timeline (i.e., more immediate relationships between emotion and perception and longer-term relationships between emotion and memory). While there were several areas that exhibited sub-regional specificity for these effects, further investigations using a more rigorous approach (e.g., anatomical ROIs) is necessary to draw strong interpretations about these findings. As such, the current discussion will focus on identified regional specificity – i.e., activity in the medial frontal, precentral, superior temporal, and middle occipital gyri, associated only with emotional distraction, and activity in the superior parietal cortex, associated only with emotional memory.

Increased activation in the medial frontal gyrus (BA 10) linked to the immediate impact of emotional distraction on perception is consistent with a large body of research showing sensitivity of this region in response to emotional stimuli (Keightley et al., 2003; Scheuerecker et al., 2007), possibly reflecting increased motivational significance of emotional stimuli (Dolcos et al., 2004a). Activity in the precentral gyrus has been reported in a number of studies of emotion processing (e.g., LaBar et al., 1998; Morris et al., 1998; Canli et al., 2002; Keightley et al., 2003; Wicker et al., 2003; Scheuerecker et al., 2007) although in most investigations this area was not the main the focus of investigation and hence typically was left out of discussion. Studies discussing its role, though, have suggested the involvement of this region is due to the motor control/imagery associated with viewing emotionally arousing stimuli (Canli et al., 2002; de Gelder et al., 2004) or with imitating emotional expressions (Lee et al., 2006). Although the superior temporal gyrus activity identified here is too inferior to be included in the temporal parietal junction (TPJ), its involvement is consistent with evidence linking activity in this region with attentional re-orienting associated with processing task-irrelevant emotional distraction(Corbetta and Shulman, 2002; Vuilleumier and Driver, 2007; Frank and Sabatinelli, 2012), and with evidence linking TPJ activity with sustained visual spatial attention (Thakral and Slotnick, 2009) toward the emotionally distracting stimuli. Lastly, increased middle occipital activity likely reflects a boost in visual processing received by emotional items linked to increased extrastriate processing mediated by both cortical-cortical and subcortical-cortical mechanisms (Vuilleumier and Huang, 2009).

Turning to the area associated only with emotional memory, it is interesting to note the effect observed in the superior parietal cortex dissociated from that identified in the inferior parietal lobe, which was present in both the immediate and long-term effects of emotional distraction. Considering these results in the framework of stimulus-driven vs. goal-directed attention networks (Corbetta and Shulman, 2002), the present results are consistent with the idea that memory benefits from both increased bottom-up contributions through inferior parietal activation (possibly reflecting capture of attentional resources) and top-down involvement from superior parietal cortex (possibly indicative of goal-relevant processing). Given that the target and distracter were contiguous and presented simultaneously, the superior parietal activity for items that were later remembered may be the result of goal-relevant processing resources being allocated to the item as a whole, and thus the emotional distracters benefited under conditions where an increase in goal-relevant resources was needed to successfully perform the task (i.e., short stimulus duration). The contribution of the superior parietal cortex to the long-term effect of emotional distraction is also consistent with event-related potential evidence that encoding processes contributing to enhanced memory for emotional events occur faster than for neutral events (Dolcos and Cabeza, 2002), presumably within a time window consistent with the present short duration. This evidence along with our findings suggest that parietal contribution to emotional memory may, in fact, be optimized under shorter exposure durations, perhaps indicating that its contribution can be more automatic than previously thought.

## Conclusion

In summary, this study provided initial evidence for a direct link between the immediate and long-term impact of emotional distraction during a lower-level perceptual task in which the to-be-remembered items were task-irrelevant. First, a direct relationship between the immediate and long-term effects of emotional distraction was identified only under conditions of limited processing resources available at encoding. Also, the engagement of mediated mechanisms, once additional resources were available, diminished the effect of the automatic mechanisms on memory. Second, consistent with a role of automatic mechanisms linking these opposing effect, AMY-HC activity was common to both the immediate/impairing effect of emotional distraction and the long-term/enhancing impact of emotion on memory. Furthermore, whereas a hemispheric disassociation was identified in AMY and HC, with both sides associated with emotional distraction and left AMY and anterior HC linked to emotional memory, a clear asymmetry was identified in the posterior HC, with only the left side contributing to successful encoding of emotional items. Third, brain regions were identified as being specifically susceptible to emotional modulation during distraction or memory formation, with activity in the medial frontal, precentral, superior temporal, and medial occipital gyri being linked to increased impact of emotional distraction, and activity in the superior parietal cortex being linked to better memory for emotional distracters. These findings demonstrate that the relationship between emotional distraction and memory is context dependent and that specific brain regions may be more or less susceptible to the direction of emotional modulation (*increased* or *decreased*), depending on the task manipulation and processes investigated. Understanding the mechanisms linking emotional distraction and memory offers important insight into clinical conditions, such as depression and anxiety, where both of these effects are dysfunctionally exacerbated.

## Conflict of Interest Statement

The authors declare that the research was conducted in the absence of any commercial or financial relationships that could be construed as a potential conflict of interest.
